# Moderate vs. Excessive Video Gaming: Distinct Neurocognitive and Neural Activation Profiles in Preadolescence

**DOI:** 10.21203/rs.3.rs-10468432/v1

**Published:** 2026-07-31

**Authors:** Bader Chaarani, Leigh-Anne Cioffredi, Matthew D. Albaugh, Nicholas Allgaier, Robert Althoff, Alexandra Potter, Hugh P. Garavan

**Affiliations:** University of Vermont - Department of Psychiatry – Burlington, VT, USA

## Abstract

The pervasive rise of video gaming in adolescent life necessitates a clearer understanding of its complex impact on cognitive and mental health development, as previous research has yielded conflicting results due to small sample sizes and unaddressed confounding factors. Here we leverage the Adolescent Brain Cognitive Development (ABCD) Study dataset (N = 9,712; mean age 9.92 years) to examine relationships between varying video gaming exposure levels and neurocognitive, behavioral, and neural outcomes. Controlling for critical environmental and demographic covariates, we show that associations between gaming and developmental outcomes are not uniform. Moderate gaming (<1 h/day) was associated with enhanced cognitive performance, including elevated fluid and total IQ, and lower externalizing and aggressive behavior scores compared to non-gamers. Conversely, excessive gaming (≥3 h/day) correlated with significantly higher attention problems and ADHD scores. Notably, all gaming cohorts outperformed non-gamers on the N-back task, with heavy gamers exhibiting significantly increased BOLD activation in the right precuneus and right middle frontal gyrus. These findings provide a comprehensive neurodevelopmental framework, supporting the hypothesis that while excessive use presents behavioral risks, moderate video gaming may be associated with enhanced cognitive and mental health profiles.

## Introduction

The world of gaming has expanded in recent years, becoming one of the most influential industries globally. As of 2024, the global community has grown to include approximately 3.32 billion active gamers and is expected to cross 3.5 billion gamers in 2025^1^. According to a recent PEW research center survey, 85% of adolescents in the U.S. play videogames^2^, a significant increase from an estimated 70% adolescent videogamers (VGs) in 2013 (AAP, 2013). This rapid expansion has motivated the scientific community to investigate the potential impact of videogaming, particularly in children and adolescents undergoing critical neurocognitive and mental health development.

For many years, a dominant perspective in scientific research has linked videogaming to a range of adverse behavioral outcomes in children. Meta-analyses have consistently pointed towards a positive correlation between playing violent video games and heightened aggressive behavior, aggressive thoughts, and aggressive feelings^3^. However, alongside these concerns, a more nuanced understanding has emerged with findings being divided with respect to videogaming’s association with cognitive skills and mental health^3^. Interestingly, a meta-analysis examining the impact of gaming during the COVID-19 pandemic found no association between gaming time and mental health, suggesting that contextual factors can significantly influence this relationship ^30^. Other research has shown that individuals who regularly play action games often demonstrate superior performance in visual attention tasks compared to those who do not^8^. Furthermore, specific cognitive skills such as mental flexibility, planning, visual and verbal working memory, and visuospatial processing have been found to be positively predicted by video game play^14^. Some evidence suggests that playing video games can be a source of stress relief and may offer benefits for adolescents experiencing anxiety, depression, ADHD, and PTSD^19^. This also aligns with other studies showing that online gaming environments can provide children with a sense of community and belonging, which is recognized as a protective factor for mental well-being^21^.

Conversely, not all research aligns with these findings. One study reported no significant links between video game playing and the cognitive abilities of young children^16^. One study found a significant association between the amount of time adolescents spent playing video games and poorer mental health, with an increased likelihood of experiencing moderate or higher levels of mental health symptoms for each additional hour of play ^28^. Moreover, the frequency of video game play has been associated with an increase in emotional problems among children ^29^. Simultaneously, excessive engagement with video games has been implicated in technology addiction, sleep disturbances, diminished academic performance, and impaired social interactions and emotional intelligence^5^. In addition, a connection has been observed between technology addiction stemming from video games and the prevalence of ADHD and anxiety among young individuals^5^.

The exploration of video games’ impact on cognitive skills reveals a complex landscape of potential benefits and conflicting results. One factor that likely drives these discrepancies is that these studies were conducted in relatively small datasets, limiting their generalizability and statistical power to detect true effects, and minimizing their capacity to investigate videogaming by different exposure levels. Additionally, while a meta-analysis suggested an overall positive association between action video game play and cognitive skills, it also identified the presence of publication bias, cautioning against definitive conclusions^9^. This suggests that studies with null or negative findings might be less likely to be published, potentially skewing the overall perception of the effects.

The majority of studies thus far evaluated video gaming activity within the context of few other variables. There is a known correlation between video game use and poor sleep outcomes^1–4^. Further, poor sleep is associated with adolescent behavioral and mental health problems. Yet few studies assess the impact of video gaming on neurobehavioral outcomes while accounting for sleep. Recently, other interpersonal factors have been associated with increased video gaming such as parental use of screen time as a reward or punishment. At the same time, increased parental monitoring and decreased family conflict have been associated with lower screen use in adolescents in the Adolescent Brain Cognitive Development (ABCD) study^^®^5,6^. The inclusion of these contextual variables will likely advance the understanding of the populations most susceptible to the impacts of video game use.

We have recently shown^3^, using the baseline assessment of the large (ABCD) study dataset, that video gaming for ≥3h/day is associated with better cognitive performance in children (ages 9–10) but scores on dimensional measures of psychopathology, including attention problems and depressive symptomatology, albeit well below clinical thresholds, compared to non-videogamers (NVGs). Here, we investigate these effects across all video gaming levels in analyses that include measures of sleep and the family environment. We hypothesize that there may be a threshold at which adolescents experience the cognitive benefits of video gaming without the increase in behavioral symptomatology.

## Methods

### Sample

Participants were enrolled in the ongoing longitudinal ABCD Study. The ABCD sample was largely recruited through public, private, and charter elementary schools. The ABCD study adopted a population neuroscience approach to recruitment by using epidemiologically informed procedures to ensure demographic variation in its sample that would mirror the variation in the US population of 9- and 10-year-olds^1^. A probability sampling of schools was conducted within the defined catchment areas of the study’s nationally distributed set of 21 recruitment sites in the US. All 9–10 year-old children in each sampled school were invited to participate after classroom-based presentations, distribution of study materials, and telephone screening for eligibility. Exclusions included common MRI contraindications (such as cardiac pacemakers and defibrillators, internal pacing wires, cochlear and metallic implants, and Swan-Ganz catheters), inability to understand or speak English fluently, uncorrected vision, hearing or sensorimotor impairments, history of major neurologic disorders, gestational age less than 28 weeks, birth weight less than 1200 g, birth complications that resulted in hospitalization for more than 1 month, current diagnosis of schizophrenia, moderate or severe autism spectrum disorder, history of traumatic brain injury, or unwillingness to complete assessments.

The study data were from the 5.1 data release. The ABCD study’s de-identified data, including all assessment domains, are released annually to the research community. Information on how to access ABCD study data through the NIH Brain Development Cohorts (NBDC) data sharing platform is available on https://www.nbdc-datahub.org/. The ABCD Study recruited 11,874 healthy children, ages 9–10, to be followed into early adulthood. The ABCD study was approved by the appropriate institutional review boards: most ABCD research sites rely on a central Institutional Review Board at the University of California, San Diego for the ethical review and approval of the research protocol, with a few sites obtaining local IRB approval. This study was carried out in accordance with the ethical guidelines for journal publications put forth by the Committee on Publication Ethics (COPE) and International Committee of Medical Journal Editors (ICMJE). Participants provided written assent, and their legal guardians written consent, for participation. The child’s age and sex were reported by the parent at the baseline assessment.

### Screen Time Survey

Participants were administered a screen time survey that asked how much time they spend engaged in different types of screen time on a typical weekday and a typical weekend day. The different screen time categories were as follows: “Watch TV shows or movies?”; “Watch videos (such as YouTube)?”; “Play video games on a computer, console, phone, or other device (Xbox, PlayStation, iPad)?”; “Text on a cell phone, tablet, or computer (eg, GChat, Whatsapp, etc.)?”; “Visit social networking sites like Facebook, Twitter, Instagram, etc?”; and “Video chat (Skype, Facetime, etc)?” For each of these activities, the participants responded with how much time they spent per weekday and weekend day doing them. They could answer none, less than 30 minutes, 30 minutes, 1 hour, 2 hours, 3 hours, or 4 hours. For each participant, a total weekly video-gaming score was derived as the sum of (video-gaming hours per weekday × 5) + (video-gaming hours per weekend day × 2). Using the video-gaming score, we defined five samples consisting of NVGs who never played video games (0 gaming hours per week) and children who play <1h/day, 1–2h/day, 2–3h/day and ≥3h/day.

### Youth mental health problems

Mental health symptoms were assessed using the Child Behavior Checklist (CBCL) [@achenbach2000], a parent-report measure consisting of 113 items rated on a 3-point scale ranging from 0 (Not True) to 2 (Very True or Often True). In the ABCD Study, these data were obtained via a self-administered, computerized version of the measure. Specifically, the CBCL yields dimensional assessments of common emotional and behavioral problems in children and adolescents. In the present study, we used raw scores from the broadband Internalizing and Externalizing scales, as well as the empirically derived and DSM-oriented subscales.

### Family environment and sleep problems

Confounders included family conflict scores, which were derived from the conflict subscale of the family environment scale (Moos and Moos, 1976), and selected to assess one of the core aspects of social interaction and family climate (Zucker et al., 2018). An extensive literature has indicated that these characteristics are directly relevant to the development of externalizing behaviors, leading to interpersonal difficulties and ultimately to the impairment of a successful adaptation to adult life (Glatz et al., 2020, Loukas et al., 2001). In addition, parental monitoring scores were included, derived from the Parental Monitoring Questionnaire, to assess parental oversight of youth behavior, capturing aspects like supervision and awareness of a child’s activities. Finally, total sleep hours were derived from the Sleep Disturbance Questionnaire in the ABCD Study, a 45-item tool evaluating sleep behaviors over the past week, generating eight subscales (e.g., bedtime resistance, sleep duration, night wakings) and a total sleep disturbance index.

### Neurocognitive performance

Neurocognitive performance was assessed using the NIH Toolbox^®^ cognition battery (see http://www.nihtoolbox.org), administered via iPad and consisting of seven different tasks that cover episodic memory, executive function, attention, working memory, processing speed, and language abilities (Bleck et al., 2013; Gershon et al., 2013a; Hodes et al., 2013). The Toolbox^®^ was normed for samples between the ages of 3 and 85 years. The total administration time for the NIH Toolbox^®^ Cognitive battery is approximately 35 min. Despite the availability of a Spanish language version (Casaletto et al., 2016; Flores et al., 2017), the ABCD study administers only the English language version (Casaletto et al., 2015) to youth given that English fluency is an inclusion criterion. The Toolbox Picture Vocabulary Task^®^ (Gershon et al., 2014, 2013b) measures language skills and verbal intellect. The Toolbox Oral Reading Recognition Task^®^ is a reading test that asks individuals to pronounce single words. The Toolbox Pattern Comparison Processing Speed Test^®^ (Carlozzi et al., 2013, 2014; Carlozzi et al., 2015) is a measure of rapid visual processing. The Toolbox List Sorting Working Memory Test^®^ requires participants to use working memory to sequence task stimuli based on category membership and perceptual characteristics. The Toolbox Picture Sequence Memory Test^®^ was modeled after memory tests asking children to imitate a sequence of actions using props (Bauer et al., 2013; Dikmen et al., 2014). The Toolbox Flanker Task^®^, a variant of the Eriksen Flanker task (Eriksen and Eriksen, 1974), is a response inhibition/conflict monitoring task that measures the ability to modulate responding under congruent versus incongruent stimulus contexts. The Toolbox Dimensional Change Card Sort Task^®^ measures cognitive flexibility (Zelazo et al., 2013, 2014). Each of the Toolbox^®^ tasks produces a number of scores, some of which are adjusted based on participant demographics. For each task, raw scores, uncorrected standard scores, and age-corrected standard scores are available (Casaletto et al., 2015). Age-corrected standard scores from this toolbox were used in the analyses.

### fMRI N-back task

The N-back task was selected from the ABCD imaging battery to probe working memory. Participants practiced the task prior to scanning to ensure that they understood the instructions and were familiar with the response collection device. The ABCD imaging protocol was designed to extend the benefits of high temporal and spatial resolution of imaging protocols of the Human Connectome Project^18^ with the multiple scanner systems of participating sites^19^. In this working memory block-design task, participants saw a series of stimuli and indicated whether each one was the same or different from the stimulus N items earlier (i.e., “N back”). The task included two conditions: a 2-back as the active condition and a 0-back as the baseline condition, which included similar visuo-motor demands but lower working memory load. In the 0-back condition, participants indicated if each stimulus matched a single target presented at the beginning of the trial, thereby obviating the need to maintain and update a two-item working memory load throughout the task. Responses on the 2-back and 0-back were input on a two-button keypad, with one button indicating the stimulus was a match and the other indicating no match (see Figure 2-b). The EN-back consisted of two runs, each containing eight blocks of trials and four 15 sec rest periods containing just a fixation cross. Blocks contained 10 trials lasting 2.5 sec each and were preceded by a 2.5 sec instruction screen indicating the condition for the upcoming block. Of the 10 trials in each block, 2 were targets, 2–3 were non-target lures, and the remainder were non-lures (i.e., stimuli only presented once). There were 160 trials in total with 96 unique stimuli of 4 different stimulus types (24 unique stimuli per type). Three-quarters of the stimuli types were human faces, demonstrating happy, fearful, or neutral facial expressions, with facial expression stimulus type held constant within each block. Contrasts of interest include 0-back vs. fixation, 2-back vs. fixation and 2-back vs. 0-back.

To assess behavioral task performance on the task, D’ was computed for both the 2-back and 0-back conditions by calculating each participant’s hit rate, the proportion of targets for which the participant correctly indicated a match, and false alarm rate, the proportion of non-targets for which the participant incorrectly indicated a match or did not respond. The hit and false alarm rates were then z-transformed. D’ was calculated as the z-transformed hit rate minus the z-transformed false alarm rate. Children were excluded from the analyses if D’ was less than 0.

After applying exclusion criteria for poor image quality, motion, or task performance^19^, the sample size available for baseline assessment was 6,009 for the N-back task. Extensive details of the task and the robust activation patterns of its main contrast are available in our previous article^20^.

### Participant Inclusion Criteria

Participants were included if they had complete information on the two videogaming screentime questionnaire items (weekday hours & weekend hours), and for all other non-imaging variables. Participants were excluded if they reported illogical gaming hours ( >22h/day). For the N-back analyses, participants were excluded if they failed the imaging quality control, which requires having 1) two fMRI runs per task, 2) hemispheric mean beta-weights within three standard deviations of the sample mean for each task, 4) at least 200 degrees of freedom over the two scan runs, 5) mean framewise displacement < 0.9 mm for both runs.

### Statistical Analyses

Outcomes of interest included mental health and behavioral scores from the CBCL, cognitive scores from the NIH Toolbox^®^ cognition battery, in addition to working memory performance and cortical BOLD signal from the N-back fMRI task (available for approximately half the sample), as described above. The cortical BOLD signal was calculated in regions of interest (ROIs) defined by the Destrieux parcellation atlas^7^ for three contrasts: 0-back vs. fixation, 2-back vs. fixation, and 2-back vs. 0-back. For each contrast, only ROIs deemed active for the task were analyzed. An ROI was considered active if its activation effect size, measured by Cohen’s D, was 0.2 or greater. Cohen’s D was computed the mean of the ROI beta-weights (activation) divided by their standard deviation.

These outcomes were compared across the NVG and VG groups using linear mixed-effects (LME) models comparing each VG group to the reference NVG group. To control for potential confounds, a set of covariates including age, sex, race/ethnicity, combined parental income, TV watching, parental monitoring, family conflict, sleep hours, and physical activity as nuisance covariates, were included in the models, in addition to scanner site as a random effect. FDR-corrected *p* values<.05 were considered significant. All analyses were coded in Python 3.11, and the statsmodels library was used to run the LMEs.

## Results

### Demographics

**Figure 1.**
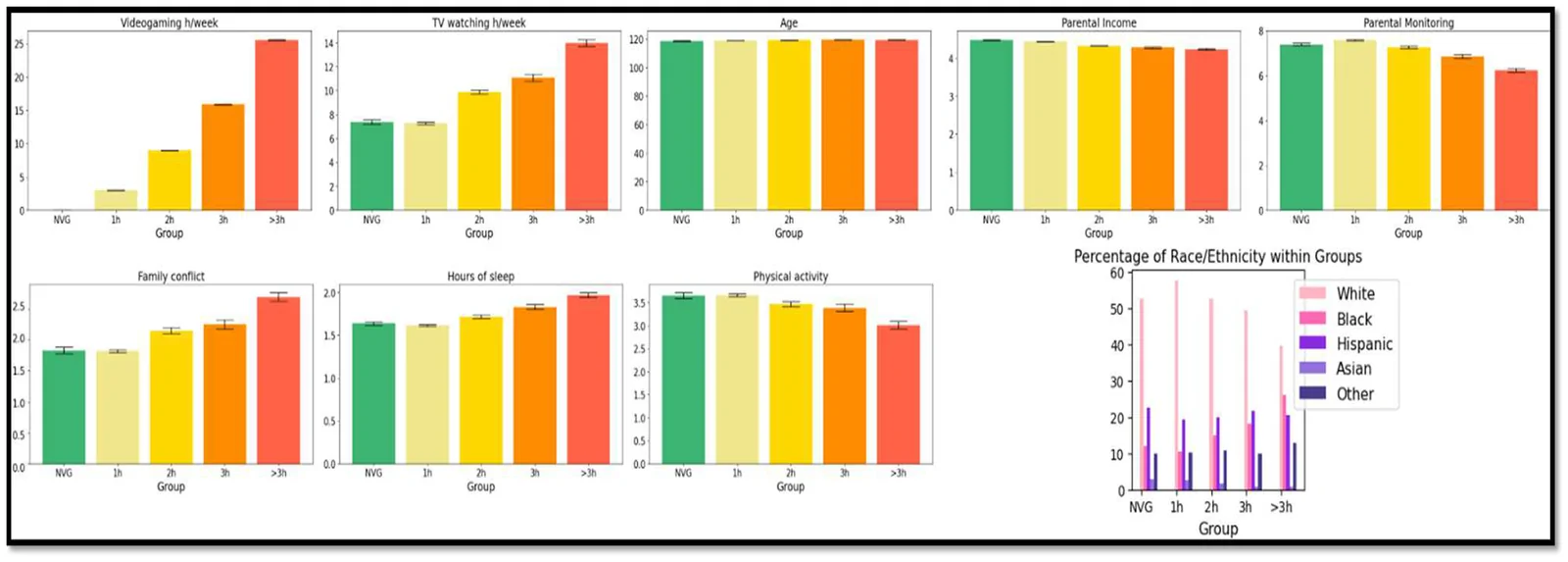
Graphic representation of the demographics and confounders (raw means) across non-videogamers (NVG) and all videogamer groups.

The final sample consisted of 9712 children: 1425 NVGs and 8287 VGs. The VG groups were composed of n=4766 children who played less than one hour per day, n=1821 who played 1–2 hours, n=802 who played 2–3 hours, and n=898 who played three or more hours per day (Table 1). Raw means for the demographics and confounding factors across non-videogamers (NVG) and all videogamer groups (<1h, 1–2h, 2–3h, >3h) are detailed in Table 1 and graphically represented in Figure 1.

### Associations of confounder variables and videogaming

ANOVA and Chi-Square tests were used to test for differences for each confounder variable across all groups. Age remained relatively stable across groups (118.67 to 119.59 months; η^2^=0.001; p=0.035), while TV watching increased with videogame use (7.38 to 13.99 h/week; η^2^=0.088; p<0.001). Parental income (η^2^=0.029; p<0.001) and monitoring (η^2^=0.024; p<0.001) showed slight decreases as videogame use rose. Family conflict was associated with higher use (η^2^=0.02; p<0.001), alongside reductions in sleep hours (η^2^=0.018; p<0.001) and physical activity (η^2^=0.007; p<0.001). As videogame use increased, the percentage of females decreased (Cramer’s V=0.33; p<0.001) and racial distribution varied significantly across groups (Cramer’s V=0.08; p<0.001), with notable shifts in proportions. All of the confounding variables were included as covariates in the subsequent analyses.

### N-back task

**Figure 2.**
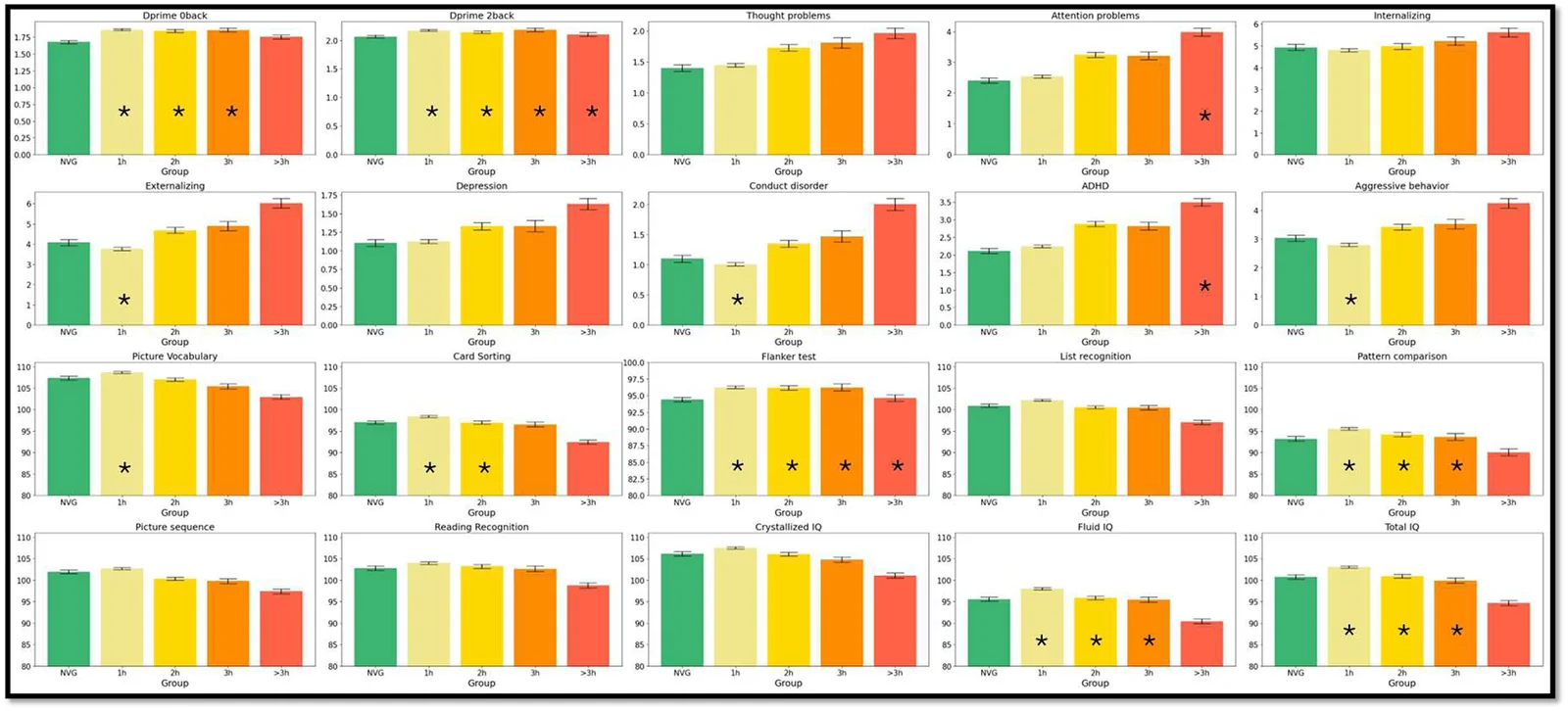
N-back behavioral performance measures, CBCL and NIH toolbox Cognitive task scores (raw means) across non-videogamers (NVG) and all videogamer groups at the baseline assessment. Asterisks indicate significant differences between NVG and videogamers with FDR-corrected *p*<.05.

All VGs groups (<1h/day: n=4306; 1–2h/day: n=1631; 2–3h/day: n=711; >3h/day: n=750) performed significantly better than the NVG group (N=1278) on the N-back task during 0-back and 2-back as measured by D-prime (FDR *p*<.05) (Figure 2 - top row) (Table 2).

**Figure 3.**
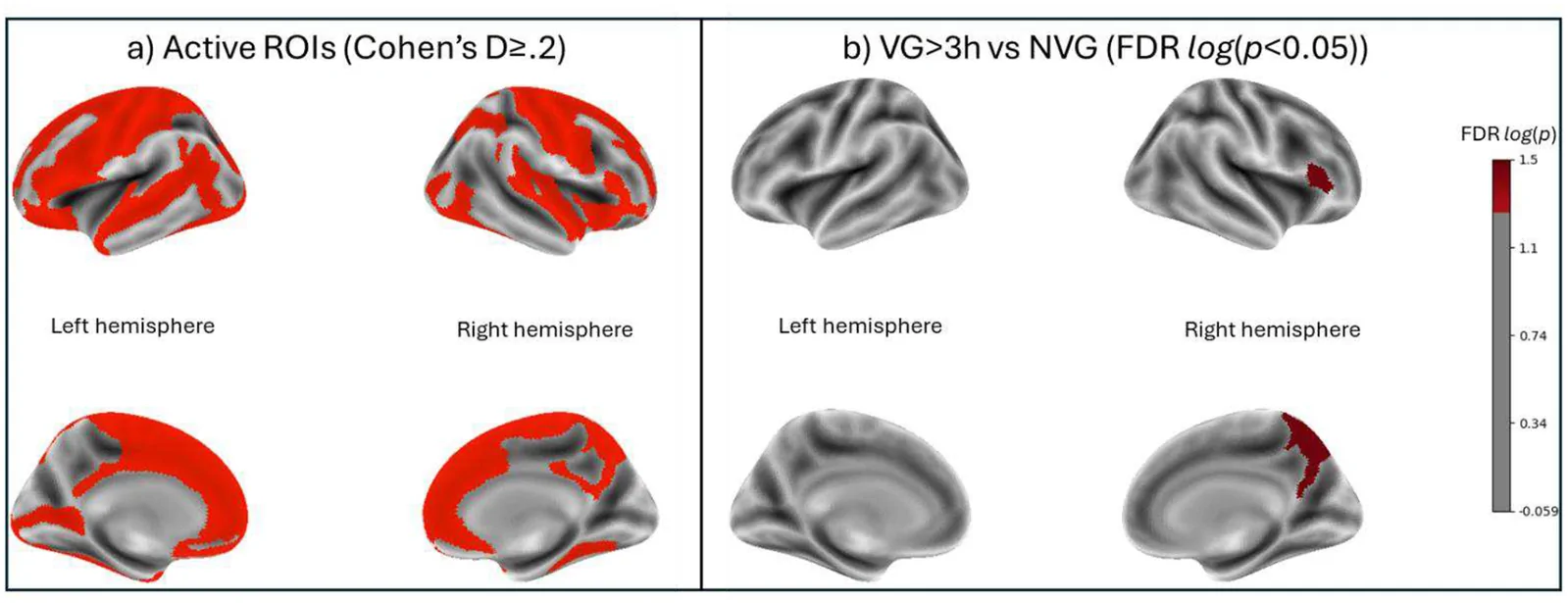
2-back vs. 0-back results. a) Active Regions of Interest (ROIs) in red with Cohen’s D≥.2. b) Significant BOLD differences (red areas) between non-videogamers (NVG) and videogamers who play 3h/day in the right precuneus and right middle frontal gyrus (FDR-corrected *p*<.05).

The BOLD activation analysis identified 114 out of 148 ROIs as active for the 2-back vs. 0-back contrast, with Cohen’s d ≥ 0.2 and Bonferroni-corrected p<0.05 (Figure 3a). LME analyses of these 114 ROIs revealed significantly greater BOLD activation in VGs playing >3h/day compared to NVGs in the right precuneus (β= 0.0377; SE=0.0114; FDR p=0.0335; 95% CI: 0.0154–0.06; Cohen’s d=0.47) and right middle frontal gyrus (β=0.0431; SE=0.0125; FDR p=0.033; 95% CI: 0.0186–0.0676; Cohen’s d=0.52), after FDR correction at p<.05 (Figure 3b) (Table 2). No BOLD differences were detected between NVG and the other VG groups or in other contrasts after FDR correction at *p*<.05. All these results would hold in a simpler model, only including age and sex as nuisance covariates in addition to scanner site as a random effect.

### Mental health and behavioral outcomes

Analysis of mental health outcomes revealed positive associations between weekly hours of video gaming and both attention problems (β=0.44; SE=0.156; FDR p=0.023; 95% CI: 0.134–0.746) and ADHD scores (β= 0.447; SE=0.134; FDR p=0.008; 95% CI: 0.185–0.709), with VGs who played >3h per day demonstrating significantly higher scores on these measures compared to the NVGs (Figure 2 - top rows) (Table 2). On the other hand, interestingly, VGs who played 1h or less/day had significantly lower externalizing (β=−0.473; SE=0.173; FDR *p* < .015; 95% CI: −0.812 to −0.134), conduct disorder (β=−0.186; SE=0.069; FDR *p* < .015; 95% CI: −0.321 to −0.052), and aggressive behavior (β= −0.344 ;SE=0.13; FDR *p* = .016; 95% CI: −0.598 to −0.089) scores than the NVG group (Table 2). All these results would hold in a simpler model, only including age and sex as nuisance covariates in addition to scanner site as a random effect.

### NIH Toolbox tasks

Overall, VGs were the best performers on the NIH toolbox cognitive tasks compared to NVGs (Figure 2 *- bottom rows)*. LME showed that all VG groups scored significantly better on the Flanker task, and all VGs who played 3h or less/day scored significantly better than NVG on pattern recognition, fluid IQ and total IQ (FDR *p*<.05) (Table 2). Importantly, VGs who played 1h or less/day scored higher than all groups on all tasks and had higher scores on picture vocabulary (β=1.261; SE=0.504; FDR *p*=0.022; 95% CI: 0.274–2.249) and card sorting (β=1.599; SE=0.948; FDR *p*=0.002; 95% CI: 0.659–2.54) tests compared to NVGs.

All these results would hold in a simpler model, only including age and sex as nuisance covariates in addition to scanner site as a random effect.

## Discussion

There is much interest in understanding the impact of video games on child development. The literature thus far demonstrates correlations between adolescent video gaming and increased mental health difficulties, while cognitive improvement has also been demonstrated. There are studies indicating increased video gaming is associated with detriments to adolescent’s social lives while others report social benefits. Thus far, the majority of the studies using ABCD data have evaluated screen media use and time video gaming as continuous variables^8–10^. Yet historical data suggest there may be a benefit from small amounts of media use in adolescence^11,12^ therefore, comparing effects of gaming in a categorical manner may reveal information that might? otherwise be missed. In fact, the American Academy of Pediatrics has long acknowledged the potential benefits of moderate screen media use while recognizing the many risks. Specifically, current AAP screen time recommendations for families and adolescents include developing a family media plan, avoiding access to media in children’s bedrooms, and focusing on getting recommended physical activity and sleep^13,14^.

This is the first study to our knowledge that includes assessments of both adolescent sleep habits and the family environment in addition to TV watching when assessing the relationship between video gaming and mental health and cognitive outcomes. Consistent with the literature, higher levels of video gaming were associated with higher family conflict, lower parental monitoring, and fewer hours of sleep reported. The mixed-effects models demonstrate the potential importance of including these measures in assessments of the impacts of video gaming behaviors as differences in sleep and parental monitoring both had significant effects with relatively large beta weights. Importantly, while accounting for differences in individual sleep patterns, and the family environments, small amounts of screen time were associated with improved scores on cognitive measures while spending more than 3+ hours daily playing video games was associated with increased attention problem scores.

The current study takes advantage of the large sample of adolescents in the ABCD Study and aimed to evaluate the impact of the quantity of time spent video gaming on emotional and behavioral outcomes as well as cognitive outcomes, and specifically compares both those who never play video games to those with mild, moderate and heavy video game use. The results support the hypothesis that video gaming associations depend, at least in part, on the quantity of time spent gaming. Our findings replicate a larger scale study investigating the links between screen time use and mental health in 120,115 15-years old English adolescents^15^. In that study, the authors concluded that videogaming for less than 1 or 2h/day was not intrinsically harmful and was associated with improved mental well-being compared to NVGs. Both studies strongly support the “Goldilocks hypothesis” as it applies to video gaming, suggesting that the relationship between time spent gaming and positive outcomes follows an inverted U-shaped curve. While this previous study accounted for sex, ethnicity, and economic status, our analysis further controlled for TV watching, site, family conflict, sleep disturbances, parental monitoring and physical activity. Additionally, we extend the findings by demonstrating associations with BOLD signal changes in cortical regions in the brain. This model posits that moderate engagement is more beneficial than either no engagement or excessive engagement. We observed this pattern clearly in both cognitive and behavioral domains. Specifically, consistent with the peak of this curve, VGs who play moderately for less than 1h/day were found to be the best performers on the NIH Toolbox cognitive tasks outperforming both NVG and VGs who play for extended hours. Interestingly, this ‘just righ’ effect extended to mental health, where moderate VGs had no or dramatically fewer behavioral problems. Conversely, as gaming time increased, the benefits diminished and detriments emerged, illustrating the downward slope of the curve. This was evident in our finding that heavy VGs had significantly higher scores for attention problems and ADHD compared to non-gamers, highlighting the potential negative consequences associated with the upper end of the gaming spectrum.

Further, the enhanced performance on the N-back task in VGs is supported by previous studies showing that VG outperform NVG on a range of cognitive tasks^16^(a flanker task, an enumeration task, and two attentional blink tasks), and on crystallized and fluid intelligence measures assessed via the NIH Toolbox^^®^ 17^. In addition, supporting our findings, research on videogame training in groups of NVG using action videogames (mainly enhancing one’s attentional control) demonstrated that videogame training consistently led to transferrable improvements in cognitive performance^18^. Our N-back analyses in the 2-back vs. 0back contrast, specifically probing working memory capacity (Baddeley, 2003), revealed significantly greater BOLD activation in VGs engaging in ≥3 hours/day of gaming compared to NVGs in the right precuneus and right middle frontal gyrus. These findings align with prior research suggesting that video gaming may enhance neural activity in the precuneus, implicated in visuospatial processing and attention (Cavanna & Trimble, 2006), which may reflect heightened spatial awareness developed through gaming. Similarly, increased activation in the middle frontal gyrus, a key region linked to decision-making and cognitive control (Talati & Hirsch, 2005), suggests that prolonged gaming may support executive processes. The enhanced BOLD activation in the precuneus and middle frontal gyrus in video gamers playing ≥3 hours/day may reflect compensatory neural recruitment or hyperactivation due to prolonged gaming, potentially indicating enhanced neural efficiency in visuospatial and executive functions, yet with possible overactivation that does not yield proportional behavioral benefits.

Alternatively, these BOLD changes might represent neuroplastic adaptations specific to gaming, such as increased sensitivity to game-related stimuli, rather than directly correlating with cognitive or mental health outcomes.

This study has important limitations to acknowledge. First the data we use for measurements of exposures, in particular time playing video games, is self-reported by youth. These subjective reports may be imperfect. There are limited data validating self-reported measures of screen time in youth. Previous reports however suggest parental estimates of screen time may be similarly flawed. Thorne et al indicate only 11% of parents/youth (8–10 years) dyads demonstrated concordant estimates of screen time^20^. In a few studies evaluating objective mobile phone app monitoring vs youth reported screen time, it appears that in later adolescence youth are relatively accurate at reporting their screen activity studies^21^. However, evaluation of screen use in younger children who are less likely to use a personal mobile device is methodologically more difficult. Further, these studies do not track video game use that occurs on consoles other than a mobile phone. That said, if data from studies such as the one we report are to be considered to guide clinical practice, it may be more important to assess exposures in a similar manner to that which a clinician would receive (patient reported behaviors) to guide recommendations. A second important limitation is the lack of granularity of video game characteristics that are played by young adolescents in the ABCD study. Video games come in several genres and modalities such as single player, multiplayer, strategy, sport, shooter, puzzle and role-playing games. These in turn could have differing impacts on adolescent mental health and cognition^22^. This depth of assessment is not available in ABCD, however future studies could investigate the possibility of differing impacts based on video game genre. In spite of these limitations the current study adds meaningful information to the current understanding of the impact of video game use in early adolescence.

In our prior research^19^, VGs playing three or more hours daily displayed less activation in visual processing areas, such as the occipital cortex and calcarine sulcus, alongside higher activation in cognitive control regions, including the cingulate, frontal gyri, and precuneus. In the present study, we successfully replicated the heightened activation in the precuneus and frontal gyri but did not observe less activation in visual areas. This partial replication likely stems from two methodological changes: first, we utilized LME models incorporating additional covariates rather than permutation testing; second, we analyzed regions of interest (ROIs) instead of cortical vertices. Notably, reduced activation in the visual cortex and calcarine sulcus among VGs playing three or more hours daily was observed when we excluded the additional covariates (family conflict, sleep duration, physical activity and parental monitoring) from the analyses, consistent with our previous findings. This suggests that these covariates may partially account for the reduced activation in the visual areas which may not be solely attributable to gaming duration. For example, sleep duration could influence visual processing efficiency (Przybylski et al., 2017).

Overall, this study provides a new perspective on the complex relationship between video gaming, cognitive skills, and mental health outcomes in children. The findings suggest that video gaming, in moderation, may offer cognitive benefits with no or dramatically fewer detrimental associations with mental health and behavioral problems. Nevertheless, caution should continue to be exercised with excessive video gaming. It also replicates the findings that characteristics of the family environment and adolescent sleep habits are associated with cognitive and mental health outcomes^23–27^. Together, the findings align with current American Academy of Pediatrics recommendations to emphasize healthy sleep and physical activity behaviors and develop family plans about screen time use.

## Figures and Tables

**Table 1.** Raw means of the demographics and confounder variables across non-videogamers (NVG) and all videogaming groups. Parental income and monitoring, family conflict, sleep hours and physical activity scores are in arbitrary units. Higher sleep hours scores reflect less sleep hours. ANOVA and Chi-Square tests were used to compare differences of the variables across videogaming groups. Eta-squared and Cramer’s V values are reflective of effect sizes of ANOVA and Chi-Square tests, respectively.

Group/Variable	Videogaming (h/week)	Age (months)	TV watching (h/week)	Parental income	Parental Monitoring	Family conflict scale	Sleep hours	Physical activity	Sex (% Female)	Race (% by category)
NVG (n=1425)	0 (0)	118.67 (7.55)	7.38 (6.91)	7.4 (2.39)	4.49 (0.5)	1.83 (1.96)	1.64 (0.77)	3.65 (2.33)	78.20%	1: 52.8%, 2: 12.1%, 3: 22.8%, 4: 3.0%, 5: 9.9%
VG<1h (n=4766)	3 (1.53)	119.08 (7.52)	7.26 (6)	7.59 (2.23)	4.44 (0.47)	1.82 (1.83)	1.62 (0.77)	3.66 (2.27)	54.30%	1: 57.6%, 2: 10.6%, 3: 19.3%, 4: 2.5%, 5: 10.2%
VG 1–2h (n=1821)	8.97 (1.95)	119.21 (7.48)	9.88 (7.30)	7.27 (2.32)	4.33 (0.51)	2.15 (2.01)	1.72 (0.81)	3.47 (2.3)	33.20%	1: 52.8%, 2: 14.9%, 3: 20.1%, 4: 1.8%, 5: 10.9%
VG2–3h (n=802)	15.9 (1.8)	119.59 (7.59)	11.09 (7.86)	6.86 (2.47)	4.28 (0.51)	2.25 (2.02)	1.83 (0.86)	3.38 (2.3)	29.90%	1: 49.6%, 2: 18.3%, 3: 21.7%, 4: 0.9%, 5: 10.0%
VG>3h (n=898)	25.56 (2.85)	119.45 (7.42)	13.99 (9.2)	6.25 (2.58)	4.24 (0.59)	2.69 (2.04)	1.97 (0.87)	3.01 (2.39)	24.00%	1: 39.7%, 2: 26.1%, 3: 20.5%, 4: 0.9%, 5: 12.9%
Test		ANOVA	ANOVA	ANOVA	ANOVA	ANOVA	ANOVA	ANOVA	Chi-Square	Chi-Square
Effect size		*η*^2^=0.001	*η*^2^=0.088	*η*^2^=0.029	*η*^2^=0.024	*η*^2^=0.02	*η*^2^=0.018	*η*^2^=0.007	Cramer’s V=0.33	Cramer’s V=0.08
P-value		0.035	<.001	<.001	<.001	<.001	<.001	<.001	<.001	<.001

**Table 2.** Statistical results from linear mixed-effects models comparing videogaming groups (categorized by hours per day) to a reference non-videogaming group. Beta coefficients, standard errors (SE), uncorrected P-values, and FDR-corrected P-values are presented for differences in mental health scores, NIH Toolbox cognitive task performance, and N-back performance and BOLD activation in significant regions of interest. Results with uncorrected P-values>.05 are omitted for simplicity. MFG: middle frontal gyrus.

Outcome measure	Videogaming Group	Beta	SE	P-value	FDR P-value	95% Cl Lower	95% Cl Upper
Aggressive behavior	1h	−0.344	0.13	0.008	0.016094	−0.598	−0.089
Attention problems	>3h	0.44	0.156	0.005	0.023865	0.134	0.746
ADHD	2h	0.227	0.107	0.034	>0.05	0.018	0.437
	>3h	0.447	0.134	0.001	0.00823	0.185	0.709
Conductdisorder	1h	−0.186	0.069	0.007	0.01488	−0.321	−0.052
Depression	>3h	0.191	0.091	0.036	>0.05	0.013	0.368
Externalizing	1h	−0.473	0.173	0.006	0.01488	−0.812	−0.134
card sorting	1h	1.599	0.48	0.001	0.002457	0.659	2.54
	2h	1.422	0.582	0.015	0.041509	0.282	2.562
	3h	1.49	0.725	0.04	>0.05	0.07	2.911
Flanker test	1h	1.508	0.429	0	0.00147	0.667	2.349
	2h	2.067	0.52	0	0.00071	1.047	3.086
	3h	2.414	0.648	0	0.00196	1.144	3.684
	>3h	1.91	0.65	0.003	0.022033	0.636	3.185
Pattern comparison	1h	2.955	0.688	0	0.000113	1.607	4.303
	2h	3.369	0.834	0	0.00071	1.735	5.003
	3h	3.516	1.038	0.001	0.002828	1.481	5.551
Picture vocabulary	1h	1.261	0.504	0.012	0.022327	0.274	2.249
Reading recognition	3h	2.1	0.87	0.016	0.044966	0.396	3.805
Total IQ	1h	1.95	0.508	0	0.000492	0.955	2.945
	2h	1.867	0.615	0.002	0.00802	0.661	3.073
	3h	2.38	0.767	0.002	0.006383	0.877	3.883
Fluid IQ	1h	2.582	0.526	0	9.00E-06	1.552	3.613
	2h	2.235	0.637	0	0.00228	0.985	3.484
	3h	2.714	0.794	0.001	0.002828	1.157	4.27
D-prime 0-back	1h	0.134	0.027	0	9.00E-06	0.081	0.186
	2h	0.117	0.032	0	0.002093	0.053	0.181
	3h	0.147	0.041	0	0.002007	0.067	0.226
	>3h	0.091	0.041	0.028	>0.05	0.01	0.172
D-prime 2-back	1h	0.119	0.029	0	0.000195	0.062	0.176
	2h	0.121	0.035	0.001	0.002436	0.052	0.19
	3h	0.179	0.044	0	0.0011	0.092	0.267
	>3h	0.158	0.045	0	0.00823	0.069	0.247
2-back vs 0-back right MFG	>3h	0.0431	0.0125	0.0006	0.0335	0.0186	0.0676
2-back vs 0-back right precuneus	>3h	0.0377	0.0114	0.0009	0.0335	0.0154	0.06

## Data Availability

The ABCD Study’s Curated Annual Release 6.0 used in this work is available to those who have completed the process to gain access (//https://www.nbdc-datahub.org) to the ABCD Data Repository. Shared data may change in ongoing updates.

## References

[1] Hartstein L. E. The impact of screen use on sleep health across the lifespan: A National Sleep Foundation consensus statement. Sleep Health 10, 373–384 (2024). 38806392 10.1016/j.sleh.2024.05.001 PMC13181348

[2] PEW research center, May 2024, “Teens and Video Games”, https://www.pewresearch.org/internet/2024/05/09/teens-and-video-games-today/

[3] Hamre R., Smith O. R. F., Samdal O. & Haug E. Gaming Behaviors and the Association with Sleep Duration, Social Jetlag, and Difficulties Falling Asleep among Norwegian Adolescents. Int. J. Environ. Res. Public. Health 19, 1765 (2022). 35162788 10.3390/ijerph19031765 PMC8834670

[4] Dibben G. O. Adolescents’ interactive electronic device use, sleep and mental health: a systematic review of prospective studies. J. Sleep Res. 32, e13899 (2023). 37029099 10.1111/jsr.13899 PMC10909457

[5] Nagata J. M. Associations between media parenting practices and early adolescent screen use. Pediatr. Res. 97, 403–410 (2025). 38834780 10.1038/s41390-024-03243-y PMC11626836

[6] Al-shoaibi A. A. A. Family conflict and less parental monitoring were associated with greater screen time in early adolescence. Acta Paediatr. apa.17349 (2024) doi:10.1111/apa.17349. PMC11464194 39031509

[7] Destrieux C., Fischl B., Dale A. & Halgren E. Automatic parcellation of human cortical gyri and sulci using standard anatomical nomenclature. NeuroImage 53, 1–15 (2010). 20547229 10.1016/j.neuroimage.2010.06.010 PMC2937159

[8] Nagata J. M. Prospective association between screen use modalities and substance use experimentation in early adolescents. Drug Alcohol Depend. 266, 112504 (2025). 39612721 10.1016/j.drugalcdep.2024.112504 PMC11784702

[9] Nagata J. M. Screen time and mental health: a prospective analysis of the Adolescent Brain Cognitive Development (ABCD) Study. BMC Public Health 24, 2686 (2024). 39370520 10.1186/s12889-024-20102-x PMC11457456

[10] Nagata J. M. Contemporary screen time modalities and disruptive behavior disorders in children: a prospective cohort study. J. Child Psychol. Psychiatry 64, 125–135 (2023). 35881083 10.1111/jcpp.13673 PMC9771898

[11] Bélanger R. E., Akre C., Berchtold A. & Michaud P.-A. A U-Shaped Association Between Intensity of Internet Use and Adolescent Health. Pediatrics 127, e330–e335 (2011). 21242218 10.1542/peds.2010-1235

[12] Turel O. & Bechara A. Little video-gaming in adolescents can be protective, but too much is associated with increased substance use. Subst. Use Misuse 54, 384–395 (2019). 30654698 10.1080/10826084.2018.1496455

[13] COUNCIL ON COMMUNICATIONS AND MEDIA. Media Use in School-Aged Children and Adolescents. Pediatrics 138, e20162592 (2016). 27940794 10.1542/peds.2016-2592

[14] Reid Chassiakos Y. L. Children and Adolescents and Digital Media. Pediatrics 138, e20162593 (2016). 27940795 10.1542/peds.2016-2593

[15] Przybylski A. K. & Weinstein N. A Large-Scale Test of the Goldilocks Hypothesis: Quantifying the Relations Between Digital-Screen Use and the Mental Well-Being of Adolescents. Psychol. Sci. 28, 204–215 (2017). 28085574 10.1177/0956797616678438

[16] Green C. S. & Bavelier D. Action video game modifies visual selective attention. Nature 423, 534–537 (2003). 12774121 10.1038/nature01647

[17] Walsh J. J., Barnes J. D., Tremblay M. S. & Chaput J.-P. Associations between duration and type of electronic screen use and cognition in US children. Comput. Hum. Behav. 108, 106312 (2020).

[18] Green C. S. & Bavelier D. Learning, attentional control and action video games. Curr. Biol. CB 22, R197–R206 (2012). 22440805 10.1016/j.cub.2012.02.012 PMC3461277

[19] Chaarani B. Association of Video Gaming With Cognitive Performance Among Children. JAMA Netw. Open 5, e2235721 (2022). 36279138 10.1001/jamanetworkopen.2022.35721 PMC9593235

[20] Thorn J. E., DeLellis N., Chandler J. P. & Boyd K. Parent and Child Self-Reports of Dietary Behaviors, Physical Activity, and Screen Time. J. Pediatr. 162, 557–561 (2013). 23058292 10.1016/j.jpeds.2012.08.031

[21] Zhao Y. Examining measurement discrepancies in adolescent screen media activity with insights from the ABCD study. Npj Ment. Health Res. 4, 15 (2025). 40346141 10.1038/s44184-025-00131-z PMC12064680

[22] Palaus M., Marron E. M., Viejo-Sobera R. & Redolar-Ripoll D. Neural Basis of Video Gaming: A Systematic Review. Front. Hum. Neurosci. 11, (2017). 10.3389/fnhum.2017.00248 PMC5438999 28588464

[23] Gruber R. Short sleep duration is associated with poor performance on IQ measures in healthy school-age children. Sleep Med. 11, 289–294 (2010). 20156702 10.1016/j.sleep.2009.09.007

[24] Turan O. Fitbit-measured sleep duration in young adolescents is associated with functional connectivity in attentional, executive control, memory, and sensory networks. SLEEP zsaf088 (2025) doi:10.1093/sleep/zsaf088. 40156904 PMC12671439

[25] Yang X. Short sleep duration and daytime outdoor activities effects on adolescents mental health: A stress susceptibility-recovery model analysis. J. Affect. Disord. 382, 428–437 (2025). 40274127 10.1016/j.jad.2025.04.085

[26] DeVille D. C. Prevalence and Family-Related Factors Associated With Suicidal Ideation, Suicide Attempts, and Self-injury in Children Aged 9 to 10 Years. JAMA Netw. Open 3, e1920956 (2020). 32031652 10.1001/jamanetworkopen.2019.20956 PMC7261143

[27] Keller A. S. Caregiver monitoring, but not caregiver warmth, is associated with general cognition in two large sub-samples of youth. Dev. Sci. 26, e13337 (2023). 36305770 10.1111/desc.13337 PMC11090251

